# Axonal dying back of upper motor neurons in human ALS

**DOI:** 10.21203/rs.3.rs-8545414/v1

**Published:** 2026-01-12

**Authors:** Haley Cropper, Fozia Mir, Jianguo Liu, Vidushi Srivastava, Mohammed Ramizuddin, Kylie Kopecky, Ebony Mocanu, Fabien Dachet, Qin Li Jiang, Madhu Soni, Tibor Valyi-Nagy, Diana Mnatsakanova, Charles Abrams, Fei Song, Jeffrey Loeb

**Affiliations:** University of Illinois at Chicago; University of Illinois at Chicago; University of Illinois at Chicago; University of Illinois at Chicago; University of Illinois at Chicago; University of Illinois at Chicago; University of Illinois at Chicago; University of Illinois at Chicago; University of Illinois at Chicago; Rush University Medical Center; University of Illinois at Chicago; University of Illinois at Chicago; University of Illinois at Chicago; University of Illinois at Chicago; University of Illinois at Chicago

**Keywords:** amyotrophic lateral sclerosis, corticospinal tract, upper motor neuron, lower motor neuron, neuroinflammation

## Abstract

Patients with amyotrophic lateral sclerosis (ALS) present with arm, leg, or bulbar weakness with or without spasticity. While genetics plays a clear role in a subset of cases, it cannot explain why symptoms start focally or how upper (UMN) and lower motor neuron (LMN) systems are linked. Here, we examined the clinicopathological relationships between UMN and LMN disease in ten ALS patients. Detailed clinical assessments were obtained and tissues from the motor cortex, brainstem, and spinal cord were collected via a rapid autopsy protocol. Tissues were stained for UMN/LMN, myelin, axons, microglia, and pTDP43. Total RNA-sequencing was performed in the medulla, cervical, and lumbar spinal cords from each patient to identify pathways enriched at sites of disease onset. None of the patients had symptoms of frontotemporal dementia (FTD), but all had focal sites of clinical onset and spasticity, indicating both UMN and LMN involvement. Postmortem examination showed LMN degeneration and microglial activation were highest at sites of disease onset. In contrast, UMN degeneration of the corticospinal tract (CST) was present equally at all levels of the spinal cord up through the medulla, regardless of the site of disease onset. Surprisingly, there was no evidence of UMN degeneration of cortical motor neurons or their projecting axons above the brainstem. Similarly, while extensive pTDP43 aggregates were seen in degenerating LMNs, no pTDP43 aggregates were seen in UMN cell bodies or their axons. Mechanistically, RNA-sequencing implicated inflammatory pathways, especially at sites of disease onset. Our findings suggest that many ALS patients without FTD have a dying back of UMN axons, independent of the site of disease onset, which stops in the brainstem with preservation of cortical motor neurons and their proximal axons. Our findings suggest that UMN axonal degeneration can be directly triggered by LMN degeneration and inflammation.

## Introduction

Amyotrophic lateral sclerosis (ALS) is a disease leading to a progressive loss of voluntary muscle movement that involves both the upper (UMN) and lower motor neuron (LMN) systems. No cure or highly effective disease modifying therapies yet exist. Gene mutations have been identified for a small percentage of patients; this has advanced our understanding of the disease and allowed for the development of animal models [1]. However, most genetic variants show incomplete penetrance and variable age of onset, suggesting a strong environmental contribution even for cases with a genetic etiology [2].

There are a number of unresolved issues in the pathogenesis of ALS. First, while a number of hypotheses have been postulated [3], it is still unclear why ALS starts focally and how it spreads. Patients often first complain of symptoms at a focal site, which can be characterized as limb-onset when first involving an arm or leg, or bulbar-onset ALS when first involving muscles of the head/neck region. Postmortem human studies have found that LMN degeneration is most severe at sites of disease onset leading to the suggestion by Ravits et al [4, 5] that ALS starts focally and spreads contiguously up and down the ventral horn of the spinal cord. Furthermore, a small percent of ALS patients have also been found to have frontotemporal dementia (FTD) raising the possibility this disease process may often extend beyond the motor system in the brain [6].

Second, while ALS involves both UMN and LMN involvement, human clinicopathological studies of the brain and corticospinal tract (CST) are limited [7]. Little is known about the UMN system or the relationship between UMN and LMN degeneration. Third, recent studies have focused on a potential causality of the TAR DNA-binding Protein 43 (TDP-43) in ALS pathogenesis. In a variety of patients including many with FTD, TDP43 has been reported to be mislocalized from the nucleus to the cytoplasm with phosphorylated cytoplasmic aggregates (pTDP43) observed in many ALS patients, regardless of their genetic status [8–10]. However, the exact mechanism of how this leads to motor neuron degeneration has not been fully elucidated. Additionally, the differential occurrence of TDP43 aggregates on UMNs and LMNs has not been extensively studied, although it has been reported that there are more aggregates in alpha motor neurons of the spinal cord than in Betz cells of the motor cortex [11].

As part of an ongoing study to identify biomarkers and therapeutic targets to block disease progression in ALS, we collected longitudinal clinical data, including the site of onset, the presence of UMN/LMN signs/symptoms, and collected tissue from the motor cortex to the neuromuscular junction via a rapid (< 3 hour) autopsy protocol. We found that ALS patients without FTD but with clinical UMN signs, had significant site-of-onset-dependent degeneration of LMNs, yet near complete preservation of UMNs in the motor cortex. Corticospinal axons were intact down to the level of the pons; however, there was profound loss of UMN axons and neuroinflammation in the CST from the lumbar spinal cord up through the medulla regardless of onset site. Mechanistically, this suggests the possibility that LMN disease and inflammation lead to a dying back of UMN axons leading to clinical signs of UMN involvement in non-FTD ALS patients.

## Materials and Methods

### Patient recruitment, clinical data collection, and rapid autopsies

We enrolled 10 ALS patients to collect clinical data and tissues. Patients were enrolled following informed consent as approved by the UIC IRB (Protocol 2013 – 0748) according to the Declaration of Helsinki. Inclusion criteria included a confirmed ALS diagnosis and consent for imaging and/or rapid autopsy. Data stored in a REDCap database was queried for history of environmental/occupational exposures, family history, genetics, MRIs, EMGs, onset site, quantitative physical examinations of motor function, and degree of UMN/LMN symptoms. Patients had an average age of 58 years old and were 50% male. A rapid autopsy (within 3-hours) was performed collecting: primary motor cortex (left and right Brodmann Area 4 (BA4) corresponding to the face, arm, leg), brainstem (midbrain, pons, medulla), and spinal cord (cervical C4-6, thoracic T5-7, lumbar L3-5). Adjacent tissue segments from each region were fresh frozen or fixed in 4% paraformaldehyde (24 hours, 4°C), rinsed with PBS, followed by 30% sucrose and stored in optimum cutting temperature (OCT, Fisher Scientific, Hampton NH) embedding medium at −80°C.

### Tissue Staining and Immunohistochemistry

20μm sections were stained with luxol fast blue (LFB) hematoxylin and eosin (H&E) according to manufacturer instructions (Poly Scientific, Bay Shore, NY), including the motor cortex (left and right BA4 corresponding to the face, arm, leg), brainstem (midbrain, pons, medulla), and spinal cord (cervical, thoracic, lumbar). Immunohistochemical staining was performed in adjacent sections using the Vectastain Elite ABC kit (Iba1: rabbit Cat#PK6101 or NF-H: mouse Cat#PK6102, Vector Laboratories, Burlingame, CA). Microglia were stained with an antibody to the ionized calcium binding adaptor molecule-1 (Iba1 1:1000; Cat#019-19741 Wako Pure Chemical Corporation, Richman, VA) and axons with an anti-neurofilament heavy chain antibody (NF-H 1:500; Cat#RT97 Developmental Studies Hybridoma Bank, Iowa City, IA). Additional slides were stained using anti phospho-TDP-43 Ser409/410 (pTDP43; Cat#CAC-TIP-PTD-M01A, Cosmo Bio, Carlsbad, CA) together with hematoxylin as a counterstain in the motor cortex (BA4 corresponding to left arm), medulla, and cervical spinal cord. Slides were incubated with 3% H_2_O_2_ (20 minutes), permeabilized and blocked (5% serum (goat for Iba1, horse for NF-H), 5% bovine serum albumin, 0.1% TritonX in TBS) for 1 hour prior to overnight incubation with primary antibody at 4°C. Slides were incubated with secondary antibody (1:200, goat anti-rabbit for Iba1 or horse anti-mouse for NF-H) then ABC reagent for 1 hour at room temperature prior to exposure to 3’3-Diaminobenzidine tetrahydrochloride (DAB; Cat#D5905, Sigma-Aldrich, St. Louis MO) for 1 minute, and coverslipped with Cytoseal mounting medium (Epredia, Richard-Allen Scientific, Kalamazoo MI).

### Image quantification

All analyses were done in a blinded fashion. Slides were imaged on a Keyence BZ-X810 microscope. LMNs were identified histologically on H&E as large (diameter > 25μm) cells with a prominent nucleus and basophilic cytoplasm, and counted per mm^2^ for density measurements. In tissue from the *n* = 10 ALS patients, three to eight ROIs per patient in the spinal cord ventral horn or hypoglossal nucleus were quantified and plotted as individual points, for a total of: hypoglossal nuclei (*n* = 9 bulbar, *n* = 22 arm, *n* = 18 leg), cervical ventral horn (*n* = 28 bulbar, *n* = 27 arm, *n* = 13 leg), and lumbar ventral horn (*n* = 23 bulbar, *n* = 28 arm, *n* = 15 leg). Qualitative assessment of the LFB H&E staining revealed loss of myelin (pink) and areas heavily myelinated (blue). For microglia and axon quantification, three 0.4mm^2^ (20x Iba1) or 0.1mm^2^ (40x NF-H) maximum intensity projection brightfield images were acquired per region of interest. Iba1 and NF-H images were thresholded to include positive staining using the ImageJ (v1.54f) thresholding tool. The average percentage area covered of positive Iba1 staining was calculated for each patient and region. For NF-H, axons were counted using ImageJ Analyze Particles tool after thresholding. Counts per 0.1mm^2^ were averaged per patient per region. Sholl analysis was performed on single cells from thresholded Iba1 images processed [12] using the Sholl plug-in from ImageJ Neuroanatomy package. A random coordinate generator was used to randomly select 6 cells per region of interest per patient (*n* = 60 cells total per region). Sensory tracts were used as internal controls for each patient. pTDP43 quantification was performed by counting the percent of pTDP + neurons of all neurons within a randomly selected high-powered view (two 0.16mm^2^ ROIs used per subject and region, n = 20 per region). Given the variable and high background, we only counted pTDP43 + fibrillary tangles as positive staining. We routinely perform a no-primary control without the addition of the anti-pTDP43 antibody to remove false positives due to the high intrinsic background staining of postmortem human neurons.

### Total RNA-sequencing

Tissues collected from all ten patients were rapidly frozen from autopsies performed within 3 hours of death and stored at −80 degrees Celsius. Multiple thin frozen sections encompassing the entire frozen cervical, lumbar spinal cord medulla were combined and total RNA was isolated using the Qiagen RNeasy Lipid Tissue Kit (Cat#74804 Qiagen, Venlo, Netherlands) according to manufacturer’s protocol. An average of 3μg of RNA per sample was sent to the UIC Genome Research Core facility for processing. In brief, RNA was treated with DNAse (RCC-5, Zymo, Irvine CA) and RNA quality was assessed using Qubit 4.0. Only samples with RNA integrity number ≥ 7 were used. RNA library preparation was completed by the UIC Genome Research Core and samples were run on an Illumina NovaSeq 6000 via the UIC-Urbana Core Facility. The average sequencing depth was 30–40 million paired reads per sample.

FASTQ files from Illumina BaseSpace were processed with Trim Galore (v0.6.10) and mapped to the human reference genome (GRCh38.p14) using STAR aligner (v2.7.11b) with GENCODE V47 annotation. Gene-level read counts were obtained using featureCounts from the Subread package (v2.0.4). Differential expression analysis was performed in R (v4.4.1) using DESeq2 (v1.44.0). The following comparisons were used: In Medulla only, bulbar vs. non-bulbar (arm + leg) onset and in cervical spinal cord only, arm vs. non-arm (bulbar + leg) onset (*P* < 0.01). Leg-onset comparisons within the lumbar spinal cord were excluded due to insufficient power with *n* = 2 leg-onset patients. Gene ontological analyses were performed using the DAVID database [13, 14] (GO Biological Processes FAT) using pathways with a gene count cut-off of ≥ 5 and Benjamini–Hochberg FDR < 0.01 (**Supplementary Material**). Raw sequencing data (FASTQ files) have been submitted to the NCBI Sequence Read Archive (SRA) under BioProject accession PRJNA1393076. Processed files have been submitted to the GEO repository and can be found under GSE315777.

### Statistics

A one-way ANOVA with Tukey’s multiple comparisons was used for LMN density, Iba1, NF-H, and pTDP43 quantification (*n* = 10 patients). The average Iba1 percent area covered or NF-H count/0.1mm^2^ across three images were calculated for each region of interest. For Iba1 staining in the motor cortex white matter, there were no significant differences seen bilaterally corresponding to face, arm and leg regions of the homunculus. All motor cortex regions in the white matter were averaged per patient to yield the average percent area covered of Iba1 + staining. To compare microglia morphology in various regions across the CNS, a two-way ANOVA with Tukey’s multiple comparisons was used for all Sholl analyses of microglia (Iba1). In the grey matter, main effects and the interaction between onset site (bulbar, arm, leg) and distance from soma were determined in the hypoglossal nucleus, cervical ventral horn, and lumbar ventral horn (*n* = 6 cells/region for each patient, *n* = 12–24 cells per region total). A two-way ANOVA with Tukey’s multiple comparisons was also used in the white matter (*n* = 60 cells/region total) with the following comparisons: midbrain vs. each motor cortex region (left face, left arm, left leg, right face, right arm, right leg), midbrain vs. pons, pons vs. medulla, medulla vs. cervical, cervical vs. thoracic, thoracic vs. lumbar. To compare microglia Sholl in motor to sensory tracts as an internal control, two-way ANOVAs with Tukey’s multiple comparisons were used to compare the dorsal column to the lateral corticospinal tract in the cervical, thoracic, and lumbar spinal cord, and the medial lemniscus to the pyramids in the medulla. An alpha of 0.05 was used to determine significance for all staining quantification.

## Results

### LMN loss and inflammation are maximal at sites of disease onset.

**Figure 1:**
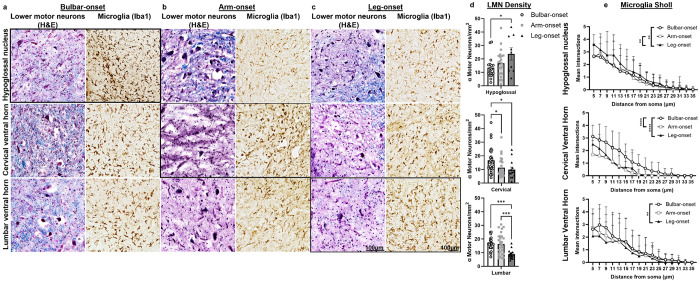
Lower motor neuron loss and associated microglial activation correspond to sites of disease onset Representative images of LFB/H&E and microglia (Iba1) staining in **(a)** bulbar-, **(b)** arm-, and **(c)** leg-onset ALS tissues show a decrease in lower motor neuron (LMN) density and increase in microglial staining corresponding to sites of onset. **(d)** Quantification of individual hypoglossal nuclei and ventral horn ROIs were plotted and consistently show a significant decrease in α-motor neuron density in the hypoglossal nucleus for bulbar- vs. leg-onset (13.5 vs. 23.8 cells/mm^2^, *P*=0.04), cervical ventral horn for bulbar- vs. arm- (16.8 vs. 11.4 cells/mm^2^, *P*=0.04) and leg-onset (16.8 vs. 9.8 cells/mm^2^, *P*=0.03), and lumbar ventral horn for leg- vs. arm- (8.9 vs. 16.3 cells/mm^2^, *P*=0.0006) and bulbar-onset (8.9 vs. 16.5 cells/mm^2^, *P*=0.0007) ALS tissues (one-way ANOVA with Tukey’s multiple comparisons, data shown as mean ± SD, *n*=3-8 images per patient/region, for a total of: hypoglossal nuclei (*n*=9 bulbar, *n*=22 arm, *n*=18 leg), cervical (*n*=28 bulbar, *n*=27 arm, *n*=13 leg), lumbar ventral horns (*n*=23 bulbar, *n*=28 arm, *n*=15 leg)). **(e)** Quantification of microglia using a Sholl analysis shows a significant difference in microglial branching in the hypoglossal nucleus for bulbar- (*P*=0.007) and arm- (*P*=0.003) vs. leg-onset, cervical ventral horn for arm- and leg- vs. bulbar-onset (*P*<0.0001), and lumbar ventral horn for leg- vs. bulbar-onset (*P*=0.004) ALS tissues (two-way ANOVA with Tukey’s multiple comparisons, data shown as mean + SD, *n*=6 cells per patient and region for a total of *n*=12-24 cells per onset and region).

The relationship between clinical disease onset and histopathology was evaluated in ten ALS patients without FTD, but with both UMN and LMN signs clinically (Table 1). All patients had sporadic ALS with an average disease duration of 4.2 years. LMN densities were significantly reduced at sites of disease onset in the hypoglossal nucleus in bulbar-onset patients (vs. leg-onset, *P* = 0.04), in the cervical ventral horn in limb-onset (arm *P* = 0.04, leg *P* = 0.03) vs. bulbar-onset, and in the lumbar ventral horn in leg-onset (vs. arm *P* = 0.0006, and bulbar *P*= 0.006) ALS patients (Fig. 1). Decreased complexity of branching is a measure of microglial activation. Sholl analysis can assesses this by quantitating the branching pattern of microglial processes. For example, microglia in the hypoglossal nucleus revealed a more activated/amoeboid morphology for bulbar- (*P*= 0.007) and arm-onset (*P*= 0.003) patients compared to leg-onset. Microglia in the cervical spinal cord were more activated/amoeboid for arm-compared to bulbar-onset (*P* < 0.0001), as well as for bulbar- compared to leg-onset (*P* < 0.0001). Lastly, microglia in the lumbar spinal cord were more activated/amoeboid for leg-onset patients compared to bulbar-onset (*P* = 0.004). These findings reinforce findings previously reported by Ravits et al [4] and implicate neuroinflammation in the degenerative process.

### UMN involvement is limited to distal upper motor axons.

**Figure 2:**
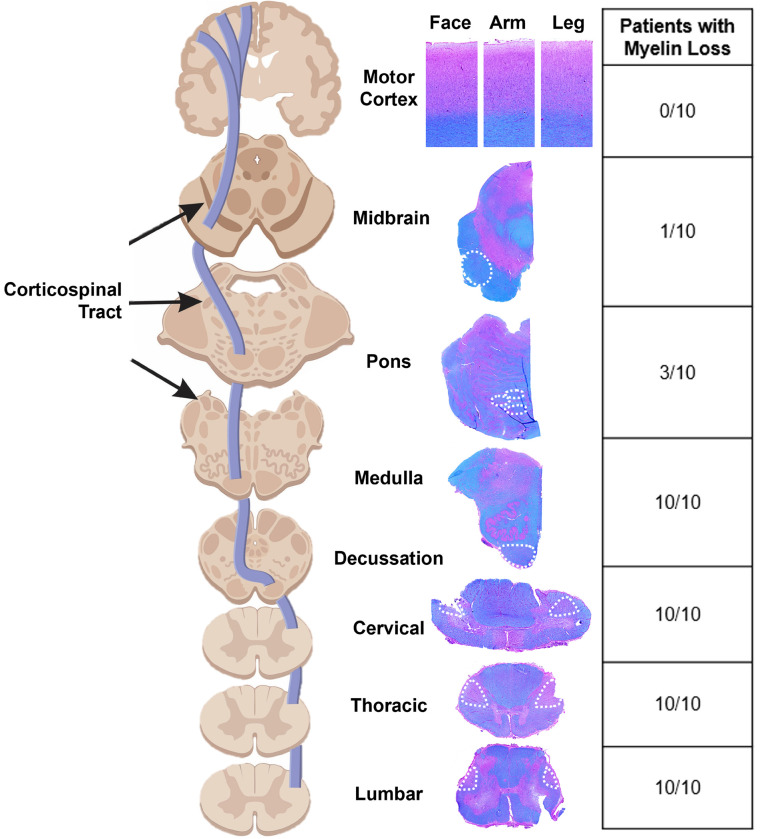
Corticospinal tract degeneration is seen at and below the medulla The left image shows the path of the CST along side sections staining from a leg-onset ALS patient using H&E/luxol fast blue (LFB) to highlight regions of myelin (blue) and myelin loss (pink) throughout the corticospinal tract from the lumbar spinal cord up through the medulla (white dashed lines). Surprisingly, there was no clear myelin loss in the pons, midbrain, and primary motor cortex (Brodmann Area 4 homunculus associated with the face, arm, and leg). The table on the right summarizes the number of patients with visible myelin loss at each level from the ten patients examined.

**Figure 3:**
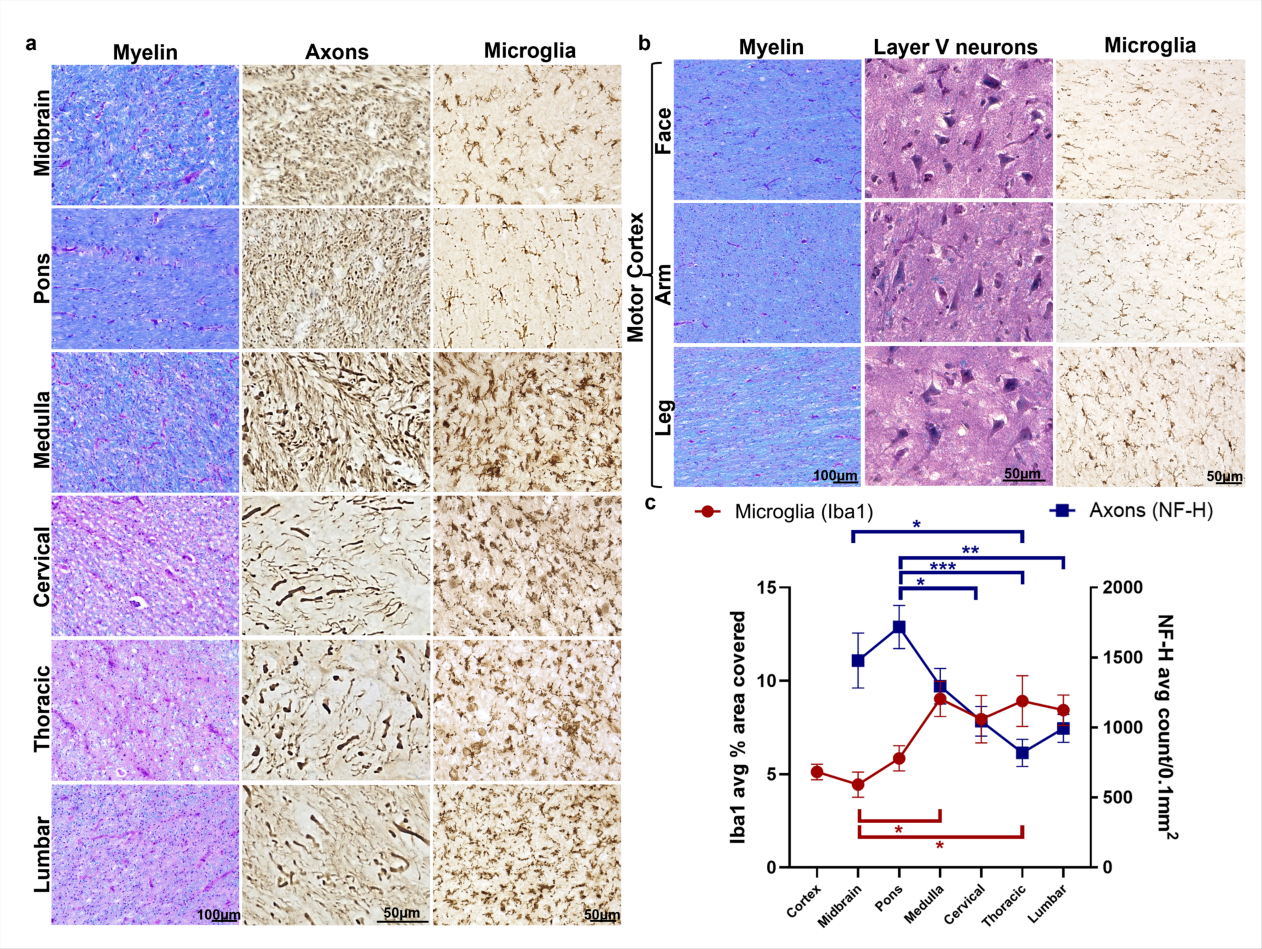
Axonal loss and microglial activation coincide with myelin loss in the corticospinal tract **(a)** Regions of the CST from a leg-onset patient showing loss of myelin (H&E/LFB) accompanied by extensive microglial activation (Iba1) and axonal loss (NF-H) in the same regions. **(b)** Primary motor cortex regions from the face, arm, and leg of Brodmann Area 4 with LFB/H&E show no myelin loss and abundant, normal-appearing layer V cortical neurons with normal microglial (Iba1) staining in the subcortical white matter. **(c)** Quantification from all ten patients of Iba1 (one-way ANOVA with Tukey’s multiple comparisons, *F*=4.22, *P*=0.001) demonstrates a significant decrease in the percent area covered of Iba1+ staining in the midbrain vs. medulla (4.43% vs. 9.03%; *P*=0.015) and midbrain vs. thoracic spinal cord (4.43% vs. 8.92%; *P*=0.020). Quantification of NF-H axon counts/0.1mm^2^ (one-way ANOVA with Tukey’s multiple comparisons, *F*=6.23, *P*=0.0001) were significantly increased in the midbrain vs. thoracic (1478 vs. 818.5 counts/0.1mm^2^; *P*=0.013), and pons (1717 count/0.1mm^2^) vs. cervical (1044; *P*=0.01), thoracic (818.5; *P*=0.0002), and lumbar (993.2; *P*=0.005) spinal cord. Data are reported as mean ± SEM (*n*=10 patients per region).

To understand the relationship between LMN loss and UMN degeneration, we examined the entire UMN system in these patients. On first pass, we found a marked loss of myelin (LFB) staining at all spinal cord levels through the medulla, regardless of site of disease onset (Fig. 2). Surprisingly, above the medulla through to the midbrain staining of the CST was almost always preserved. We also found no myelin loss in all motor cortex regions corresponding to face, arm, and leg regions bilaterally. Further analysis showed that myelin loss was accompanied by axonal loss and a marked increase in microglial activation (Fig. 3a, 3c). Neuropathological examination of the motor cortex did not reveal any clear abnormalities of layer 5/6 neurons in all areas of the homunculus bilaterally in all ten patients (Fig. 3b, 3c). The gradient of distal to proximal axon loss corresponded with microglial activation where the average microglial area was significantly higher in the thoracic spinal cord (*P*= 0.020) and medulla (*P*= 0.015) compared to the midbrain. Similarly, the axonal density was significantly lower in the spinal cord compared to the brainstem (thoracic versus midbrain (*P*= 0.013), and cervical (*P*= 0.01), thoracic (*P*= 0.0002), lumbar (*P*= 0.005) versus pons; Fig. 3c).

**Figure 4:**
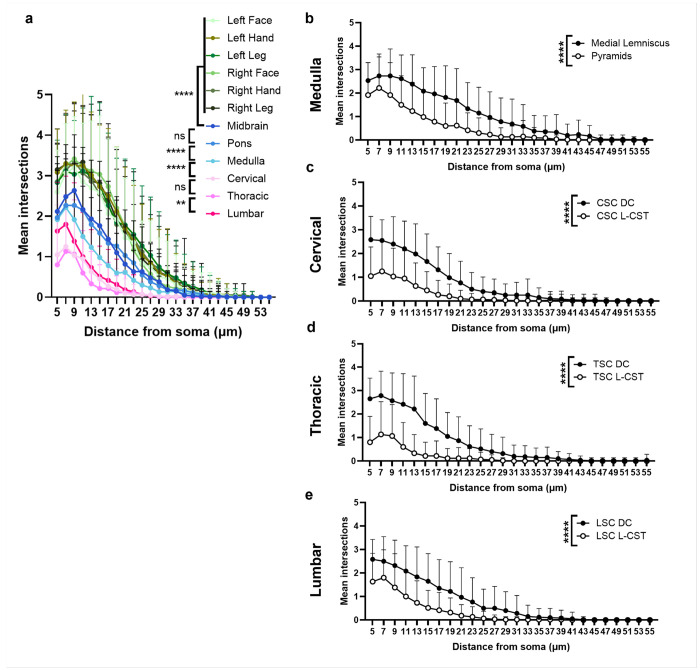
Sholl analysis shows activated microglia in only the lower portions of the corticospinal tract **(a)** Sholl analysis shows a gradient of microglial activation with less branching in more activated regions. The most ramified, resting microglia were seen in the motor cortex regions compared to those in the brainstem and spinal cord. At each level, microglial morphology in the motor tracts (pyramids and lateral-corticospinal tract (L-CST)) was compared to sensory tracts (medial lemniscus and dorsal column (DC)) as a normative control. A two-way ANOVA with Tukey’s multiple comparisons shows significant main effects of region (*F*=285.3, *P*<0.0001) and distance from soma (*F*=704.8, *P*<0.0001), as well as an interaction between the two (*F*=11.73, *P*<0.0001). There are significant differences between microglial branching in the following regions: midbrain vs. left face, left arm, left leg, right face, right arm, right leg, pons and medulla, medulla and cervical (all with *P*<0.0001), and thoracic and lumbar spinal cord (*P*=0.003). There were no significant differences in intersection points between the midbrain vs. pons (*P*>0.999) and cervical vs. thoracic (*P*=0.87). **(b-e)** Two-way ANOVAs with Tukey’s multiple comparisons show significant interactions, and main effects of distance and tract in the **(b)** medulla (*F*=7.95 interaction, *F*=111.2 distance, *F*=358.8 tract, all *P*<0.0001), **(c)** cervical (*F*=19.24 interaction, *F*=136.3 distance, *F*=397.0 tract, all *P*<0.0001), **(d)** thoracic (*F*=31.4 interaction, *F*=101.1 distance, *F*=635.1 tract, all *P*<0.0001), and **(e)**lumbar spinal cords (*F*=11.48 interaction, *F*=116.2 distance, *F*=304.1 tract, all *P*<0.0001). Data are reported as mean + SD (*n*=60 cells per region with *n*=6 cells per patient (*n*=10) and region).

Sholl analysis showed a gradient of microglial morphologies from the brain to the lumbar spinal cord. Microglia in the CST from the motor cortex through the upper brainstem had small cell bodies with more branching indicating a more normal-appearing, quiescent microglial phenotype than those in the lower brainstem and spinal cord that were larger, rounded, and phagocytic-appearing (Fig. 4a). Using sensory tracts as an internal control, we compared microglial morphology between sensory and motor tracts to confirm differences in microglial activation states. Sholl analysis revealed more ramified microglia in the sensory tracts of the spinal cord (dorsal column) and medulla (medial lemniscus), with amoeboid microglia in the motor tracts (*P*< 0.0001, Fig. 4b–e). These findings suggest a possible pathogenic role for neuroinflammation in the CST at and below the medulla.

### pTDP43 pathology was limited to LMNs.

**Figure 5:**
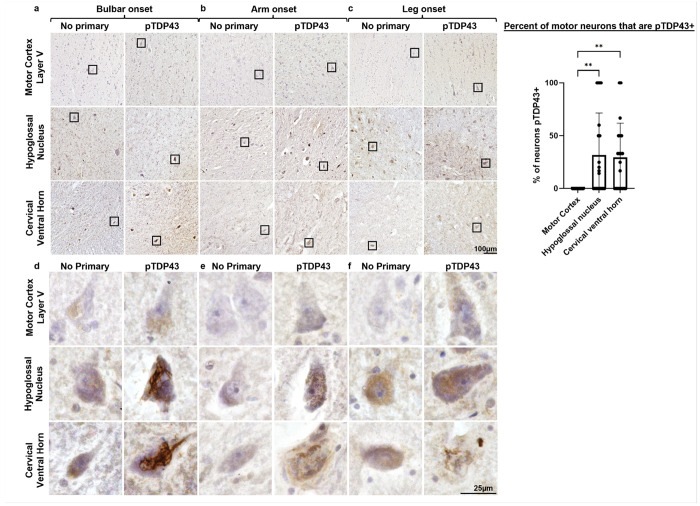
pTDP43 aggregates are seen over background staining in lower motor neurons but not in upper motor neurons. Representative images from the motor cortex (BA4 layer V corresponding to the left hand of the motor homunculus), hypoglossal nucleus, and cervical ventral horn with hematoxylin counter staining demonstrate increased pTDP43+ fibrillary tangles compared to adjacent control sections stained with no primary antibody in **(a)**bulbar-, **(b)** arm-, and **(c)** leg-onset ALS tissues. Positive staining of filamentous aggregates can be visualized in **(d-f)** lower motor neurons in the hypoglossal nucleus and ventral horn, but not in upper motor neurons of the motor cortex. **(g)** One-way ANOVA with Tukey’s multiple comparisons (*F*=7.23, *P*=0.0016) shows a significant increase in quantity of pTDP43+ aggregates in the hypoglossal nucleus (*P*=0.0034) and cervical ventral horn (*P*=0.0068) compared to motor cortex motor neurons. Notably, brown cytoplasmic staining can be observed in the no primary control tissues and therefore is not considered positive staining compared to samples stained with primary antibody. Data are reported as mean ± SD (*n*=20 per region; including *n*=2 ROIs per patient (*n*=10)).

Due to the reported prevalence of pTDP-43 pathology across ALS patients [9], we performed pTDP43 immunohistochemistry in motor cortex (BA4 corresponding to the left hand of the homunculus), medulla, and cervical spinal cord with a hematoxylin counterstain for neuronal visualization. Regardless of the site of disease onset, pTDP43 aggregates were observed in an average of 31.8% of LMNs in the hypoglossal nucleus and 29.58% of LMNs in the cervical ventral horn with no pTDP43 aggregates in the motor cortex (Fig. 5a–c). Due to high background staining in human tissues, in performing these studies we also performed a no primary antibody control staining at each level, which shows considerable non-specific staining especially in the cytoplasmic regions. Positive staining in the ventral horn was in the form of fibrillary structures throughout the cell body of degenerating motor neurons (Fig. 5d–f). Blinded quantification of randomly selected regions at each level showed a significant increase in pTDP43 aggregates in the hypoglossal nucleus (*P*= 0.0006) and cervical ventral horn (*P*= 0.006) compared to motor cortex layer V motor neurons (Fig. 5g).

### Transcriptional profiling shows an enrichment of inflammatory pathways at sites of disease onset.

**Figure 6:**
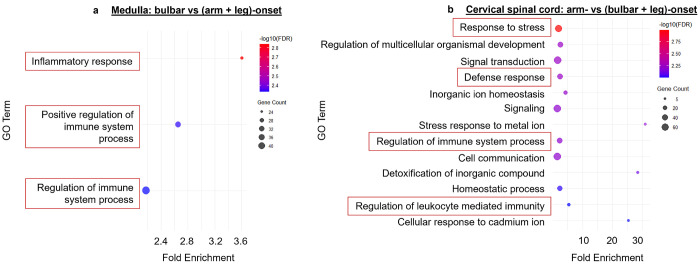
Inflammatory pathways are enriched at sites of disease onset Total RNA-sequencing from all ten ALS patients in the medulla, cervical, and lumbar spinal cord shows enrichment of gene ontology (GO) terms involving inflammatory pathways (red boxes) at sites of disease onset (*n*=4 bulbar, *n*=4 arm, *n*=2 leg). Using the DAVID online database, GO terms with a gene count ≥5 (indicated by size of circle) and an FDR<0.01 (shown as −log10FDR color gradient) were graphed by fold enrichment in tissues corresponding with the site of disease onset vs. non-onset. **(a)** In the medulla, bulbar vs. non-bulbar (arm and leg) onset shows enrichment of pathways involving inflammatory GO terms. **(b)** In the cervical spinal cord, arm vs. non-arm (bulbar and leg) onset shows enrichment of GO terms involving stress, inflammatory and defense responses.

Total RNA-sequencing was performed on entire cross sections of medulla, cervical, and lumbar spinal cord from these ten ALS patients (Fig. 6). Differentially expressed genes within the medulla and cervical spinal cord for various sites of onset were found using DESeq2 with *P*< 0.01. Enriched pathways in the medulla for bulbar- vs. non-bulbar-onset (arm + leg) patients (Fig. 6a) and cervical for arm- vs. non-arm-onset (bulbar + leg) patients (Fig. 6b) were found using the DAVID database [13, 14] (pathways with gene count ≥ 5, FDR < 0.01; **Supplementary Material**). Consistent with what we observed histologically, both comparisons predicted pathways involved with inflammation, stress, and defense. Given the low number of leg-onset patients, analysis was focused on the medulla and cervical spinal cord to determine which pathways are enriched at these sites when considering site of disease onset. Future studies will investigate specific pathways and cell clusters that are implicated at site of disease onset to identify potential therapeutic targets.

## Discussion

### LMN degeneration and microglial activation were greatest at sites of disease onset, whereas no degeneration of UMNs was observed in cortical motor neurons or their proximal axons.

ALS is a disease with clinical involvement of both LMNs and UMNs. Here we show that involvement is a far more limited for UMNs than LMNs. We were surprised to discover that while all patients had UMN clinical signs, they also had normal-appearing layer 5/6 neurons in the motor cortex with intact proximal axonal projections until they reached the lower brainstem. At and below the level of the medulla, the CST was profoundly affected with a significant loss of axons, reduction of myelin, and activation of inflammatory microglia suggesting that a dying back of distal axons from the ventral horn is responsible for UMN symptoms. Our findings, while underappreciated, are not entirely new. A study from 1941 showed the same pattern of CST neurodegeneration in a series of 37 out of 42 ALS patients [15]. CST degeneration was seen at or below the medulla in 16 out of 42 ALS patients and below the peduncle of the midbrain in 25 out of 42 ALS patients [15]. The lack of neurodegenerative changes in the motor cortex, has also been reported more recently by Coan *et al* [16] where there was only minimal loss of cortical motor neurons (Betz cells) in 76% of ALS samples they examined. While MRI studies of the CST offer additional opportunities to study the CST prior to death, results have been highly variable in the extent of CST involvement [17, 18].

Accumulation of TDP43 has been suggested as a disease mechanism common to all ALS patients and is under active investigation [8–10]. Early reports in a mix of ALS patients with and without FTD found increased TDP43 aggregates in many brain regions [19]. Focusing only on the motor cortex, we found no pTDP43 aggregates in all 10 of our patients, none of whom had FTD. In contrast, about 30% of degenerating LMNs had pTDP43 aggregates. Interestingly, we found significant background staining, especially in the cytoplasm of neurons from no-primary control immunohistochemical studies. Thus, we only considered staining above the background signal as positive staining.

Why ALS starts focally in most patients remains unclear. Risk factors for ALS include age, male sex, military service, and participation in athletics [20–23]. Anecdotally, many patients with ALS present with peripheral nerve injuries at the same site ALS onset [24–26]. We recently explored this in an animal model of ALS where a single sciatic nerve injury was sufficient to trigger ALS disease onset at that site [27, 28]. Results presented here are consistent with previous findings by Ravits et al [4, 5], that LMN degeneration at the time of death is most significantly at the sites of disease onset. However, the site of disease onset did not seem to have any effects on the degree of UMN axonal loss in the CST.

### Neuroinflammation at the site of disease onset may contribute to the subsequent spread of neurodegeneration.

Neuroinflammation has been implicated as an important pathological process and therapeutic target [28–30] in many neurodegenerative diseases. Microglial cells are known to contribute to synaptic pruning and cell death in neurodegeneration [27, 31, 32]. In the above referenced animal study, focal disease onset after peripheral nerve injury in SOD1 rodents led to significant synaptic loss prior to motor neuron cell death [27, 28]. Thus, the loss of UMN synapses associated with LMN degeneration could lead to the dying back of UMN distal axons resulting in UMN clinical symptoms.

Microglia exhibit variable phenotypes depending on activation state [33]. Resting microglia appear more ramified with smaller cell bodies and longer branching to survey the surrounding environment. Activated microglia take on an amoeboid shape with larger cell bodies and shorter branching. Sholl analyses presented here further highlights the phenotypic differences in microglial activity across the CNS in ALS tissues. Together with the RNA-sequencing results showing enrichment of inflammatory pathways at sites of disease onset, microglial responses may be critical in understanding how disease starts and spreads.

While this study is relatively small, our results are highly consistent with older literature [15, 16] and present a plausible model that links LMN to UMN degeneration in ALS through focal neuroinflammation in the ventral horn. A larger series that similarly includes detailed clinical, imaging, and pathological examinations could further support these findings.

## Supplementary Material

This is a list of supplementary files associated with this preprint. Click to download.

• SupplementaryMaterial.xlsx

## Figures and Tables

**Table 1.** Patient demographics

No.	Primary Diagnosis	Sites of Onset	Disease course (yrs)	Age (at diagnosis)	Sex
**1**	Sporadic ALS without FTD	Arm	7	32	Male
**2**	Sporadic ALS without FTD	Arm	3	59	Male
**3**	Sporadic ALS without FTD	Leg	5	44	Female
**4**	Sporadic ALS without FTD	Leg	6	57	Male
**5**	Sporadic ALS without FTD	Bulbar	3	71	Male
**6**	Sporadic ALS without FTD	Bulbar	3.5	79	Female
**7**	Sporadic ALS without FTD	Arm	5.5	49	Female
**8**	Sporadic ALS without FTD	Arm	4	56	Male
**9**	Sporadic ALS without FTD	Bulbar	1.5	59	Female
**10**	Sporadic ALS without FTD	Bulbar	3.5	70	Female

## Data Availability

All clinical data and human tissue samples are de-identified and stored in the UI NeuroRepository and available upon request following review by contacting the authors. Raw sequencing data (FASTQ files) have been submitted to the NCBI Sequence Read Archive (SRA) under BioProject accession PRJNA1393076. Processed files have been submitted to the GEO repository and can be found under GSE315777.

## Abbreviations

ALS: amyotrophic lateral sclerosis
CST: corticospinal tract
LMN: lower motor neuron
UMN: upper motor neuron
LFB: luxol fast blue
H&E: hematoxylin and eosin
Iba1: ionized calcium binding adaptor molecule 1
NF-H: neurofilament heavy chain
pTDP43: phospho-TAR DNA-binding protein 43
FTD: frontotemporal dementia
